# One‐Step Reforming of CO_2_ and CH_4_ into High‐Value Liquid Chemicals and Fuels at Room Temperature by Plasma‐Driven Catalysis

**DOI:** 10.1002/anie.201707131

**Published:** 2017-09-19

**Authors:** Li Wang, Yanhui Yi, Chunfei Wu, Hongchen Guo, Xin Tu

**Affiliations:** ^1^ Department of Electrical Engineering and Electronics University of Liverpool Liverpool L69 3GJ UK; ^2^ State Key Laboratory of Fine Chemicals School of Chemical Engineering Dalian University of Technology Dalian 116024 P. R. China; ^3^ School of Engineering University of Hull Hull HU6 7RX UK

**Keywords:** carbon dioxide, heterogeneous catalysis, methane, plasma chemistry, reforming

## Abstract

The conversion of CO_2_ with CH_4_ into liquid fuels and chemicals in a single‐step catalytic process that bypasses the production of syngas remains a challenge. In this study, liquid fuels and chemicals (e.g., acetic acid, methanol, ethanol, and formaldehyde) were synthesized in a one‐step process from CO_2_ and CH_4_ at room temperature (30 °C) and atmospheric pressure for the first time by using a novel plasma reactor with a water electrode. The total selectivity to oxygenates was approximately 50–60 %, with acetic acid being the major component at 40.2 % selectivity, the highest value reported for acetic acid thus far. Interestingly, the direct plasma synthesis of acetic acid from CH_4_ and CO_2_ is an ideal reaction with 100 % atom economy, but it is almost impossible by thermal catalysis owing to the significant thermodynamic barrier. The combination of plasma and catalyst in this process shows great potential for manipulating the distribution of liquid chemical products in a given process.

Chemical transformations of CO_2_ into value‐added chemicals and fuels have been regarded as a key element for creating a sustainable low‐carbon economy in the chemical and energy industry. A particularly significant route that is currently being developed for CO_2_ utilization is catalytic CO_2_ hydrogenation. This process can produce a range of fuels and chemicals, including CO, formic acid, methanol, hydrocarbons, and alcohols; however, high H_2_ consumptions (CO_2_+3 H_2_→CH_3_OH+H_2_O) and high operating pressures (ca. 30–300 bar) are major challenges associated with this process.

Instead of using H_2_, the direct conversion of CO_2_ with CH_4_ (dry reforming of methane, DRM) into liquid fuels and chemicals (e.g., acetic acid) represents another promising route for both CO_2_ valorization and CH_4_ activation. CH_4_ is an ideal H supplier to replace H_2_ in CO_2_ hydrogenation as CH_4_ has a high H density and is available from a range of sources (e.g., natural gas, shale gas, biogas, and flared gas). Moreover, it is an inexpensive carbon source that can increase the atom utilization of CO_2_ hydrogenation owing to the stoichiometric ratio of C and O atoms, as well as reduce the formation of water.

Recently, Ge and co‐workers investigated the direct C−C coupling of CO_2_ and CH_4_ to form acetic acid on a Zn‐doped ceria catalyst by density functional theory (DFT) modeling;^1^ this is an attractive route as the direct conversion of CO_2_ and CH_4_ into acetic acid is a reaction with 100 % atom economy [Equation (1)]. However, this reaction is thermodynamically unfavorable under practical conditions. The conventional indirect catalytic process often proceeds through two steps (Scheme 1): 1) DRM to produce syngas (CO and H_2_) at high temperatures (>700 °C), and 2) conversion of syngas into liquid fuels and chemicals at high pressures. This indirect route for CO_2_ valorization and CH_4_ activation is inefficient as the DRM process for syngas production is highly endothermic and requires high temperatures and energy input [Equation (2)]. Catalyst deactivation due to carbon deposition is another challenge impacting the use of this reaction on a commercial scale. It is almost impossible to directly convert two stable and inert molecules (CO_2_ and CH_4_) into liquid fuels or chemicals in a one‐step catalytic process bypassing the production of syngas. A stepwise method was proposed to convert CO_2_ and CH_4_ into acetic acid over Cu/Co‐based catalysts,^2^ Pd/C, Pt/Al_2_O_3_,^3^ Pd/SiO_2_, and Rh/SiO_2_ 4 by heterogeneous catalysis. The catalyst was first exposed to CH_4_, forming CH_*x*_ species on the catalyst surface. Subsequently, the feed gas was changed from CH_4_ to CO_2_, and acetic acid was formed through the reaction of CO_2_ with CH_*x*_ over the catalyst. This indirect process is complicated by the periodic change of reactants and the product collection.^5^

(1) $$\mathrm{CO}{}_{2}+\mathrm{CH}{}_{4}\to \mathrm{CH}{}_{3}\mathrm{COOH},\;\;\Delta G{}_{298\;K}=71.17\;\mathrm{kJ}\;\mathrm{mol}{}^{-1}$$

(2) $$\mathrm{CH}{}_{4}+\mathrm{CO}{}_{2}\to 2\;\mathrm{CO}+2\;H{}_{2},\;\;\Delta H{}_{298\;K}=247\;\mathrm{kJ}\;\mathrm{mol}{}^{-1}$$

**Scheme 1:**
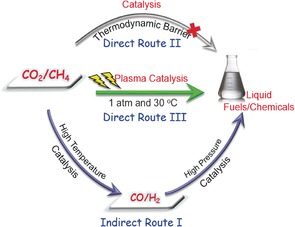
Direct and indirect processes for the conversion of CO_2_ and CH_4_ into liquid fuels and chemicals.

Non‐thermal plasmas (NTPs) offer a unique way to enable thermodynamically unfavorable chemical reactions at low temperatures owing to the non‐equilibrium character. The overall gas temperature in an NTP remains low while the generated electrons are highly energetic with a typical electron temperature of 1–10 eV, which is sufficient to activate inert molecules (e.g., CO_2_ and CH_4_) into reactive species, including radicals, excited atoms, molecules, and ions. These energetic species are capable of initiating a variety of chemical reactions. Although much effort has been devoted to the use of NTPs for the degradation of gas pollutants, far less has been done with regard to their use in the synthesis of fuels and chemicals.^6^ Previous work on DRM with NTPs mainly focused on syngas production,^7^ while very limited efforts have been devoted to the challenging one‐step conversion of CH_4_ and CO_2_ into liquid fuels and chemicals.^8^, ^9^ A few groups have reported on the formation of trace oxygenates (e.g., alcohols and acids) as side products in plasma DRM for syngas production.^10^ Thus far, the use of NTPs for the direct conversion of CO_2_ and CH_4_ into oxygenates has resulted in poor selectivities and yields.

Herein, we describe the development of a novel dielectric barrier discharge (DBD) reactor with a ground water electrode (see the Supporting Information, Schemes S1 and S2) for the one‐step conversion of CO_2_ and CH_4_ into oxygenates at room temperature (30 °C) and atmospheric pressure. This setup is unique and has not been reported previously. Figure 1 shows that no reaction occurred in the “catalyst only” mode at 30 °C without plasma. However, the use of an NTP enabled this thermodynamically unfavorable reaction to occur at room temperature and resulted in the production of liquid chemicals, including acetic acid, methanol, ethanol, and acetone, with acetic acid being the major product. Trace amounts of formic acid, propanol, and butanol were also detected in the condensed liquid. In the plasma process without a catalyst (“plasma only”), a total liquid selectivity of 59.1 % was achieved with selectivities of 33.7 %, 11.9 %, 11.9 %, and 1.6 % for acetic acid, ethanol, methanol, and acetone, respectively (Figure 1 a). The CO selectivity was only about 20.0 % (Figure 1 b), and the CH_4_ and CO_2_ conversions amounted to approximately 18.3 % and 15.4 %, respectively (Figure 1 c).

**Figure 1:**
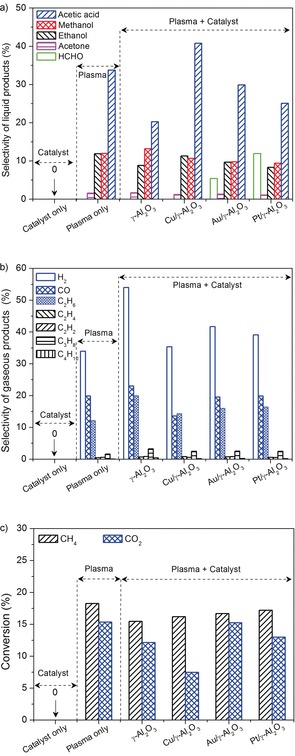
Effect of operating modes and catalysts on the reaction: a) Selectivities for oxygenates, b) selectivities for gaseous products, c) conversion of CH_4_ and CO_2_ (total flow rate 40 mL min^−1^, discharge power 10 W, catalyst ca. 2 g).

Combining the plasma process with a catalyst shows great potential for manipulating the production of different oxygenates under ambient conditions. Clearly, packing the Cu/γ‐Al_2_O_3_ catalyst in the DBD enhanced the selectivity for acetic acid to 40.2 %, compared to the plasma‐only mode and the plasma reaction using γ‐Al_2_O_3_ only (20.2 %). Acetic acid was the major product regardless of the catalyst used, followed by methanol and ethanol (Figure 1 a). HCHO was formed only when the supported noble metal catalysts were used in the plasma reaction, and the Pt/γ‐Al_2_O_3_ catalyst showed the highest selectivity to HCHO. Compared to the plasma‐only mode, placing the catalysts in the DBD gave similar gaseous product distributions, with H_2_, CO, and C_2_H_6_ being the major gaseous products (Figure 1 b). However, combining the NTP with the catalysts enhanced the H_2_ selectivity by 10–20 % (except for Cu/γ‐Al_2_O_3_), and slightly increased C_2_H_6_ production, but had a weak effect on the selectivity for CO (except for Cu/γ‐Al_2_O_3_, which decreased CO selectivity to 13.5 %) and other C_*x*_H_*y*_ (i.e., C_2_H_2_, C_2_H_4_, C_2_H_6_, C_3_H_8_, and *n*‐C_4_H_10_). In addition, compared to the plasma‐only mode, the conversion of CO_2_ and CH_4_ slightly decreased with packing catalysts. This phenomenon can be attributed to the change in discharge behavior induced by the catalyst, which had a negative effect on the reaction (Figure S1). Interestingly, C_6_H_12_O_4_ (CAS No. 49653‐17‐0) was found on the inner reactor wall in the plasma‐catalyst mode (Figure S2). These results demonstrate the feasibility of using NTPs for the direct conversion of CH_4_ and CO_2_ into higher‐value liquid fuels and chemicals in a single‐step process under ambient conditions, bypassing the formation of syngas.

To understand the formation of the liquid chemicals, optical emission spectroscopy (OES) was used to investigate the species produced in the CH_4_/CO_2_ DBD (Figure 2). H_α_ and O atomic lines and CH, C_2_, CO_2_^+^, CO_2_, and CO bands were identified in the emission spectra of the DBD, with CO, CH, and H being the major ones (Table S2).

**Figure 2:**
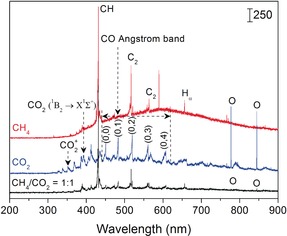
Optical emission spectra of CH_4_, CO_2_, and CH_4_/CO_2_ plasmas (total flow rate 40 mL min^−1^, CH_4_/CO_2_ ratio 1:1, discharge power 10 W, exposure time 2 s).

CO is mainly derived from reactions S1–S3 (Table S3) in the DBD. Our simulation showed that electron‐impact CO_2_ reactions produced about 95 % vibrationally excited CO_2_ (CO_2_(v)) compared to electronically excited CO_2_ as shown in Figure S3 and Table S4. O radicals generated from CO_2_ dissociation can attack CO_2_(v) molecules to produce CO (S1 and S2).^11^ Different from CH, CH_3_ derived from CH_4_ dissociation cannot be detected by OES, but recent simulations revealed that electron‐impact dissociation of CH_4_ leads to 79 % CH_3_ formation and only 15 % and 5 % CH_2_ and CH, respectively.^12^ Therefore, CH_3_ is the dominant species in the CH_4_/CO_2_ DBD. In addition to electrons (S4 in Table S3), reactive species such as OH, O, and H can also react with CH_4_ to produce CH_3_ radicals (S5–S7) in the CH_4_/CO_2_ DBD. Additionally, OH is an important species, especially for alcohol formation. In the CH_4_/CO_2_ DBD, OH could be produced indirectly by reactions S8–S13, with S8 and S9 as the major channels based on the reaction rate coefficients and *E*_a_.^13^ Special attention was given to S10, although a very low reaction rate coefficient of 1.4×10^−29^ cm^3^ molecule^−1^ s^−1^ and a high *E*_a_ value of 111 kJ mol^−1^ were determined for ground‐state CO_2_ reacting with an H radical to produce an OH radical; this reaction (S10) can be accelerated by using CO_2_(v) instead of ground‐state CO_2_,^14^ and the use of vibrationally excited reagents is most effective in overcoming the activation barrier of the endothermic reaction.^14^, ^15^ Thus the reaction CO_2_(v)+H→CO+OH could be one of the major routes for OH formation under these conditions as CO_2_ is mainly present in vibrationally excited states (Figure S3).

Based on the analysis of the gaseous and condensed liquid products and the OES results, CO, CH_3_, and OH radicals are the key species in the CH_4_/CO_2_ plasma reaction. Therefore, possible reaction pathways for the formation of acetic acid, methanol, and ethanol under these conditions are proposed in Scheme 2.

**Scheme 2:**
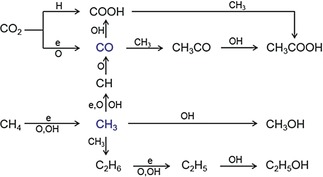
Possible reaction pathways for the formation of CH_3_COOH, CH_3_OH, and C_2_H_5_OH in the direct reforming of CH_4_ and CO_2_ with DBD.

Two possible reaction pathways could contribute to the formation of acetic acid. CO can react with a CH_3_ radical to form an acetyl radical (CH_3_CO) by reaction S14 in Table S3 with a low energy barrier of 28.77 kJ mol^−1^,^16^ followed by recombination with OH to produce acetic acid in reaction S15 with no energy barrier10g (see also Figures 3 and S4). Clearly, the selectivity to acetic acid increases initially and then decreases with the CH_4_/CO_2_ ratio, with optimal acetic acid formation at a CH_4_/CO_2_ ratio of 1:1. Correspondingly, the relative intensities of the CO band head and the O atomic line increased with a decrease in the CH_4_/CO_2_ ratio from 3:1 to 1:2 while that of the CH band head increased (Figure S4). This suggests that decreasing the CH_4_/CO_2_ molar ratio decreases the generation of CH_3_ radicals, but increases OH formation. A similar mechanism of acetic acid formation has been proposed on the basis of DFT modeling10g and by Eliasson and co‐workers.10i In addition, direct coupling of CH_3_ and carboxyl radicals (COOH) could also form acetic acid by reaction S16, while COOH radicals may be formed from reactions S17 and S18 in Table S3.10g

**Figure 3:**
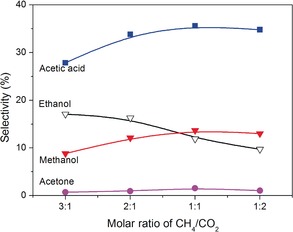
Effect of the CH_4_/CO_2_ molar ratio on the selectivity for oxygenates without a catalyst (total flow rate 40 mL min^−1^, discharge power 10 W).

Decreasing the CH_4_/CO_2_ molar ratio decreased the generation of CH_3_ radicals, but increased OH formation (Figure S4). Simultaneously, the formation of CH_3_OH increased initially with a decrease in the CH_4_/CO_2_ molar ratio and reached a peak at a CH_4_/CO_2_ molar ratio of 1:1. By contrast, the formation of C_2_H_5_OH decreased continuously as the CH_4_/CO_2_ molar ratio was decreased (Figure 3). These findings suggest that the production of CH_3_OH mainly depends on the generation of both CH_3_ and OH radicals while the formation of C_2_H_5_OH is more sensitive to the presence of CH_3_ radicals in the plasma reaction as C_2_H_5_OH formation requires twice the amount of CH_3_ radicals in comparison to the formation of CH_3_OH. As shown in Scheme 2, CH_3_OH can be directly formed from the coupling of CH_3_ and OH radicals with a high rate coefficient (S19 in Table S3),^17^ while C_2_H_5_OH formation requires several elementary reactions (S20–S24). The recombination of a CH_3_ radical with itself forms C_2_H_6_ (S20),^18^ which is followed by dehydrogenation to form a C_2_H_5_ radical by reactions S21–S23, with S21 as the primary reaction according to the reaction rates.13d, 19 The C_2_H_5_ radical finally recombines with OH to form C_2_H_5_OH with a high rate coefficient of 9.34×10^−11^ cm^3^ molecule^−1^ s^−1^ (S24).^20^

Clearly, adding catalysts to the plasma reaction influences the distribution of the formed oxygenates, especially for the formation of HCHO after addition of the Pt and Au catalysts, revealing the occurrence of surface reactions in addition to plasma gas phase reactions.^21^ In traditional catalysis, CO hydrogenation, CH_3_OH oxidation, and methylene (CH_2_) oxidation can lead to the generation of HCHO over noble‐metal catalysts.^22^ In this plasma process, adding noble‐metal catalysts in the plasma had almost no influence on the CO selectivity, but decreased the selectivity for CH_3_OH, C_2_H_5_OH, and CH_3_COOH and increased the selectivity for HCHO and C_2_H_6_ (Figure 1 a). Considering the major species that are present in the CH_4_/CO_2_ DBD, CH_*x*_ (*x*=4, 3, and 2) could be the primary source for HCHO formation by oxidation reactions. Namely, CH_*x*_ in the gas phase could be adsorbed onto the surface of the catalyst to form HCHO by the oxidation of CH_2, ad_ (CH_*x*,ad_+O, H, OH→CH_2,ad_) and to produce C_2_H_6_ by self‐recombination of CH_3_ radicals instead of converting CH_3_ into CH_3_OH, C_2_H_5_OH, and CH_3_COOH. This could explain why the presence of the Au and Pt catalysts in the plasma decreased the formation of CH_3_OH, C_2_H_5_OH, and CH_3_COOH, but enhanced the production of C_2_H_6_ and HCHO (Figures 1 a and b). Possible pathways for the formation of the major oxygenates on the catalyst surface are proposed in Scheme S3. In addition, catalyst characterization (Figures S5–S8) suggested that the metal particle size and interactions between metal and support are not determining factors for the reaction performance (Figure 1), whereas the strength of the bonding of adsorbed intermediates to the catalyst surface, that is, the oxygen adsorption energy (Δ*E*_O_), could be a good activity descriptor towards the formation of different products in DRM.^23^

In conclusion, the one‐step room‐temperature synthesis of liquid fuels and chemicals from the direct reforming of CO_2_ with CH_4_ has been achieved by using a novel atmospheric‐pressure DBD reactor. The total selectivity for liquid chemicals was approximately 50–60 %, with acetic acid as the major product. The CH_4_/CO_2_ molar ratio and the type of catalyst can be used to manipulate the production of different oxygenates. These results clearly show that non‐thermal plasmas can be used to overcome the thermodynamic barrier for the direct transformation of CH_4_ and CO_2_ into a range of strategically important platform chemicals, especially for the production of acetic acid with 100 % atom economy. Additionally, combining the DBD with noble‐metal catalysts produced formaldehyde, which cannot be generated in the same plasma reaction without a catalyst. This finding suggests that new research should be directed at designing a catalyst with high selectivity towards a desirable product.

## Conflict of interest

The authors declare no conflict of interest.

## Supporting information

As a service to our authors and readers, this journal provides supporting information supplied by the authors. Such materials are peer reviewed and may be re‐organized for online delivery, but are not copy‐edited or typeset. Technical support issues arising from supporting information (other than missing files) should be addressed to the authors.

Supplementary
