# Supplementary material for: One‐Step Reforming of CO2 and CH4 into High‐Value Liquid Chemicals and Fuels at Room Temperature by Plasma‐Driven Catalysis
Source: Angew Chem Int Ed Engl. 2017 Sep 19;56(44):13679–83. doi: 10.1002/anie.201707131 (PMC5656906; doi:10.1002/anie.201707131)
Supplement: Supplementary file 1 — Supplementary [file ANIE-56-13679-s001.pdf]

Angewandte Chemie
Eine Zeitschrift der Gesellschaft Deutscher Chemiker

Supporting Information

One-Step Reforming of CO_2 and CH_4 into High-Value Liquid Chemicals and Fuels at Room Temperature by Plasma-Driven Catalysis

*Li Wang, Yanhui Yi, Chunfei Wu, Hongchen Guo, and Xin Tu**

anie_201707131_sm_miscellaneous_information.pdf

Experimental

The experiment was carried out in a coaxial dielectric barrier discharge (DBD) reactor with a novel water electrode at atmospheric pressure and room temperature (Scheme S1). The DBD reactor consisted of a pair of coaxial glass cylinders (inner and outer glass tubes) and two coaxial electrodes. The inner high-voltage electrode was a stainless-steel rod with an outer diameter (o.d.) of 2 mm, installed along the axis of the inner glass tube (10 mm o.d. × 8 mm i.d.), which also served as the dielectric material. Compared to conventional cylindrical DBD reactor design, circulating water filled the space between the inner and outer glass cylinders and acted as a ground water electrode. This novel reactor design using the water electrode could effectively remove heat generated by the discharge and maintain the reaction at around room temperature (~30 ^oC) for the effective synthesis of liquid oxygenates at atmospheric pressure. The discharge length was 45 mm with a discharge gap of 3 mm. The catalyst was packed into the discharge area. The flow rate of CH_4 and CO_2 was controlled by mass flow controllers with a total feed flow rate of 40 ml/min. The DBD reactor was connected to an AC high voltage power supply with a maximum peak voltage of 30 kV and a variable frequency of 7-12 kHz. In this work, the frequency was fixed at 9 kHz. The electrical signals (applied voltage, current and voltage on the external capacitor) were recorded by a four-channel digital oscilloscope (Tektronix, MDO 3024). The discharge power was calculated using the Lissajous method and was fixed at 10 W in this work [1].

The dry reforming of methane (DRM) reaction was carried out at the same temperature (~30 ^oC) under three different operating modes: plasma-alone, catalysis-alone and plasma-catalysis. In the catalysis-alone mode, no reaction occurred at a temperature of around 30 ^oC.

The gaseous products were analyzed using a gas chromatograph (Shimadzu GC-2014) equipped with a thermal conductivity detector (TCD) and a flame ionized detector. A water/ice bath (0 ^oC) was placed at the exit of the reactor to condense liquid products. The oxygenates were qualitatively analyzed using a gas chromatography-mass spectrometer (GC-MS, Agilent GC 7820A and Agilent MSD 5973) and quantitatively analyzed using a gas chromatograph (Agilent 7820) equipped with a FID with a DB-WAX column. The change of the gas volume before and after the reaction was measured using a soap-film flowmeter (Scheme S1).

The emission spectra of the CH_4/CO_2 DBD were recorded using a Princeton Instruments ICCD spectrometer (SP 2758) in the range of 200-1200 nm via an optical fiber, placed near the ground electrode of the DBD reactor. The slit width of the spectrometer was fixed at 20 μm. A 300 g mm^{-1} grating was used. For comparison, the spectra of the DBD using pure CH_4 and pure CO_2 were also recorded.

Measurement of plasma reaction temperature: an infrared camera (FLIR A 40) was focused on a 9×9 mm window of the DBD reactor to measure the temperature in the discharge area, as shown in Scheme S2 (a). The results show that the reaction temperature in the discharge area was around 30 ^oC, as shown in SP1, SP2, SP3 and SP4 of Scheme S2 (b), while the temperature of the inner high-voltage electrode was slightly higher (~57 ^oC). A fiber optical thermal meter (OMEGA, FOB102) was also used to measure the temperature in the discharge area by placing the optical fiber in the discharge area. The measured temperature of the discharge area was also around 30 ^oC.

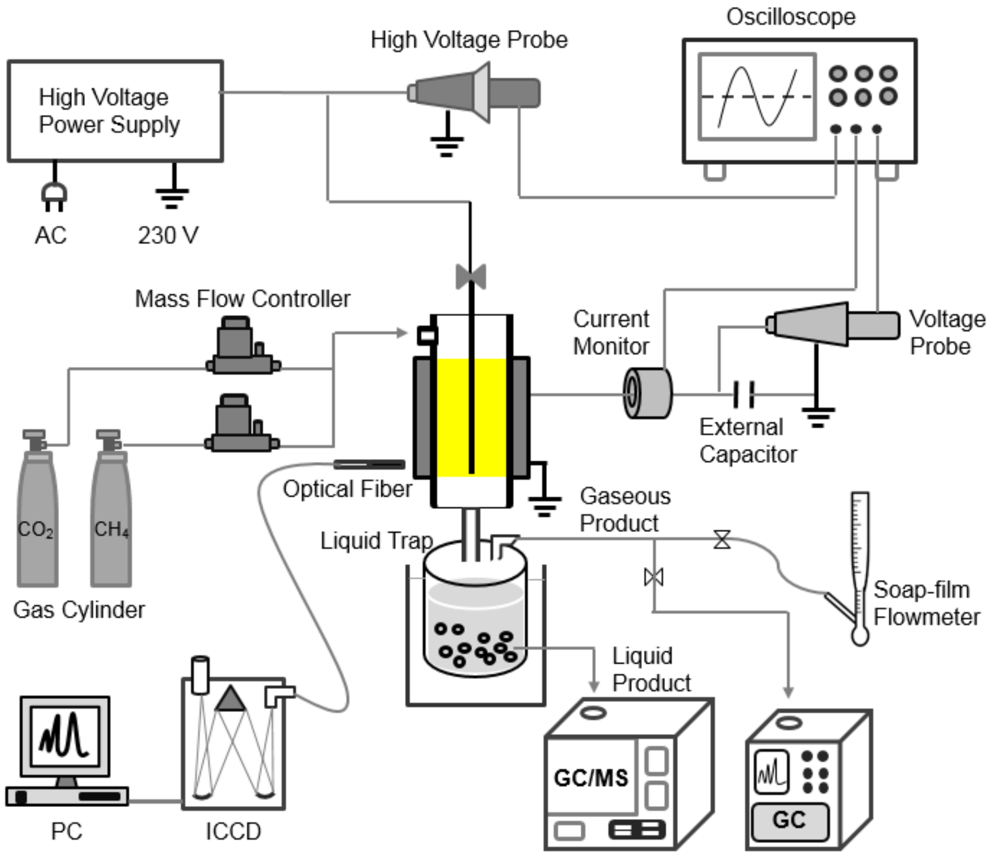

Scheme S1. Schematic diagram of experimental setup.

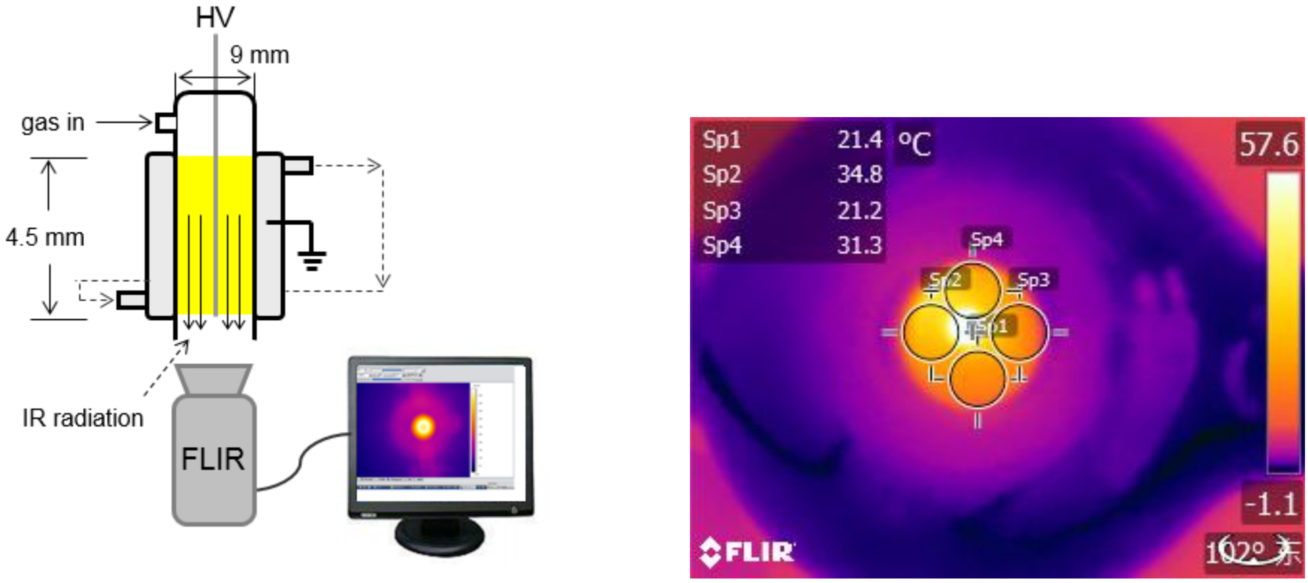

Scheme S2. Measurement of reaction temperature in the discharge area by an infrared camera.

To evaluate the performance of the dry reforming reaction, the concentration of each product in the condensate was calculated via corresponding formula of standard calibrated concentration curve (Table S1).

Table S1. Formula of standard concentration curve.

Product	Equation	Adj. R-Square	Standard Error
Acetic acid	*y* = 1.77584E-4 × *x* + 0.01268	0.99244	0.02965
Methanol	*y* = 1.60469E-4 × *x* – 0.00655	0.99456	0.00599
Ethanol	*y* = 1.28332E-4 × *x* – 0.0015	0.99955	0.00147
Formaldehyde	*y* = 0.0213 × *x* + 0.04621	0.99678	0.02554
Acetone	*y* = 8.8864E-5 × *x* – 0.00252	0.9942	0.00234

(*y* denotes as concentration of sample, mol/L; *x* denotes as GC peak area of sample).

The conversion of CH_4 and CO_2 is defined as:

$$X_{\mathrm{CH_4}}\ (\%) = \frac{\text{moles of } \mathrm{CH_4} \text{ converted}}{\text{moles of initial } \mathrm{CH_4}} \times 100 \quad (1)$$

$$X_{\mathrm{CO_2}}\ (\%) = \frac{\text{moles of } \mathrm{CO_2} \text{ converted}}{\text{moles of initial } \mathrm{CO_2}} \times 100 \quad (2)$$

The selectivity of gaseous products can be calculated:

$$S_{\mathrm{H_2}}\ (\%) = \frac{\text{moles of } \mathrm{H_2} \text{ produced}}{2 \times \text{moles of } \mathrm{CH_4} \text{ converted}} \times 100 \quad (3)$$

$$S_{\mathrm{CO}}\ (\%) = \frac{\text{moles of CO produced}}{\text{moles of } \mathrm{CH_4} \text{ converted} + \text{moles of } \mathrm{CO_2} \text{ converted}} \times 100 \quad (4)$$

$$S_{\mathrm{C}_x\mathrm{H}_y}\ (\%) = \frac{x \text{ moles of } \mathrm{C}_x\mathrm{H}_y \text{ produced}}{\text{moles of } \mathrm{CH_4} \text{ converted} + \text{moles of } \mathrm{CO_2} \text{ converted}} \times 100 \quad (5)$$

Note that the change of the gas volume before and after the reaction was taken into account in the calculation of above parameters.

The selectivity of the liquid products can be calculated according to:

$$\text{The total selectivity of liquid products } (\%) = 100\% - \left(\mathrm{S_{CO}} + \mathrm{S_{C_xH_y}}\right) - \text{ca. } 10\% \text{ carbon deposition} \quad (6)$$

The selectivity of $C_xH_yO_z$ can be calculated:

$$S_{\mathrm{C}_x\mathrm{H}_y\mathrm{O}_z}\ (\%) = \text{carbon of } \mathrm{C}_x\mathrm{H}_y\mathrm{O}_z\ (mol\ \%) \text{ in the liquid product} \times (6) \quad (7)$$

Catalyst preparation

All the catalysts were synthesized by incipient wetness impregnation over as-is commercially obtained γ-Al_2O_3 (Dalian Luming Nanometer Material Co., Ltd.) and as-synthesized TS-1 using a hydrothermal method. Metal precursor solution was prepared by dissolving each metal salt in water, which is just sufficient to fill the pores of 8 g of the corresponding support. The supports were first calcined to remove the impurities (e.g., adsorbed H_2O) in a muffle furnace at 400 ^{o}C for 5 h, then the support was added to the as-prepared precursor solution and was stirred until it was thoroughly mixed. The resulting mixture was successively kept at room temperature for 3 h, vacuum freeze-dried overnight at -50 ^{o}C and dried in air at 120 ^{o}C for 5 h. The dried sample was finally calcined in an Ar-DBD plasma at 350 ^{o}C for 3 h. Metal loading amounts of noble (Pt and Au) and non-noble metal (Cu) catalysts were ca. 1 wt.% and ca. 15 wt.%, respectively.

Catalyst characterization

The acidity of the supports was evaluated by NH_3 temperature-programmed desorption (NH_3-TPD) using a Quantachrome ChemBET 3000 Chemisorption instrument. The sample (140 mg) was pretreated at 600 ^oC for 1 h in a He flow (20 ml/min) and then cooled to 150 ^oC. The pre-treated sample was saturated with NH_3 for 30 min, and then purged with a He flow for 1 h at 150 ^oC. The TPD profile was recorded, heating the sample from 150 to 600 ^oC at a constant heating rate of 14 ^oC/min in a He flow.

The metal-support interaction was studied by H_2 temperature-programmed reduction (H_2-TPR) using the same instrument as NH_3-TPD. The sample (100 mg) was pretreated at 500 ^oC for 1 h in a He flow (20 ml/min), and then cooled to 50 ^oC. The pre-treated sample was exposed to a H_2/He mixture (10 vol.% H_2) and was heated from 150 to 800 ^oC at a constant heating rate of 14 ^oC/min to create the TPR profile.

N_2 physisorption was performed at 77 K using a Micrometrics TriStar 2020 instrument. Prior to the N_2 physisorption measurements, the samples were degassed at 350 ^oC for 3 h. The specific surface area of the samples was calculated using the Brunauer-Emmett-Teller (BET) equation.

X-ray diffraction (XRD) patterns were collected using a Rigaku D-Max 2400 X-ray diffractometer with Cu K_α radiation. Transmission electron microscopy (TEM) was used to characterize the formation of metal particles on the catalyst surface using a JEOL 2010 with EDS of Oxford Instruments INCA energy system at an accelerating voltage of 200 kV.

Results and Discussion

In this study, we found that packing the catalysts into the discharge slightly decreased the conversion of CO_2 and CH_4. Packing the catalyst pellets into the entire discharge area was found to change the discharge mode from a typical strong filamentary microdischarge in the gas phase to a combination of weak spatially-limited microdischarge and a predominant surface discharge on the catalyst, as shown in Figure S1. Similar phenomenon has also been found in our previous studies [2]. This physical effect (e.g. weak microdischarges) induced by the presence of the catalyst could affect the DRM reaction and lead to the decreased conversion of CH_4 and CO_2. Similar negative effect from the integration of plasma and catalysts has also been reported from other groups [3].

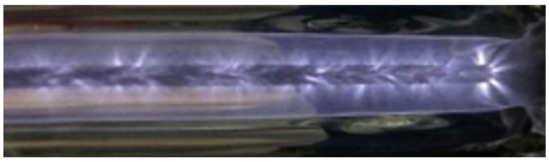

Plasma alone

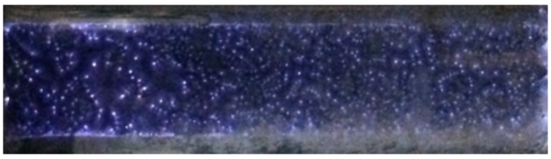

Plasma + Catalyst

Figure S1. Optical image of the discharge with and without a catalyst (total flow rate 40 ml/min, CH_4/CO_2 ratio 1:1, discharge power 10 W).

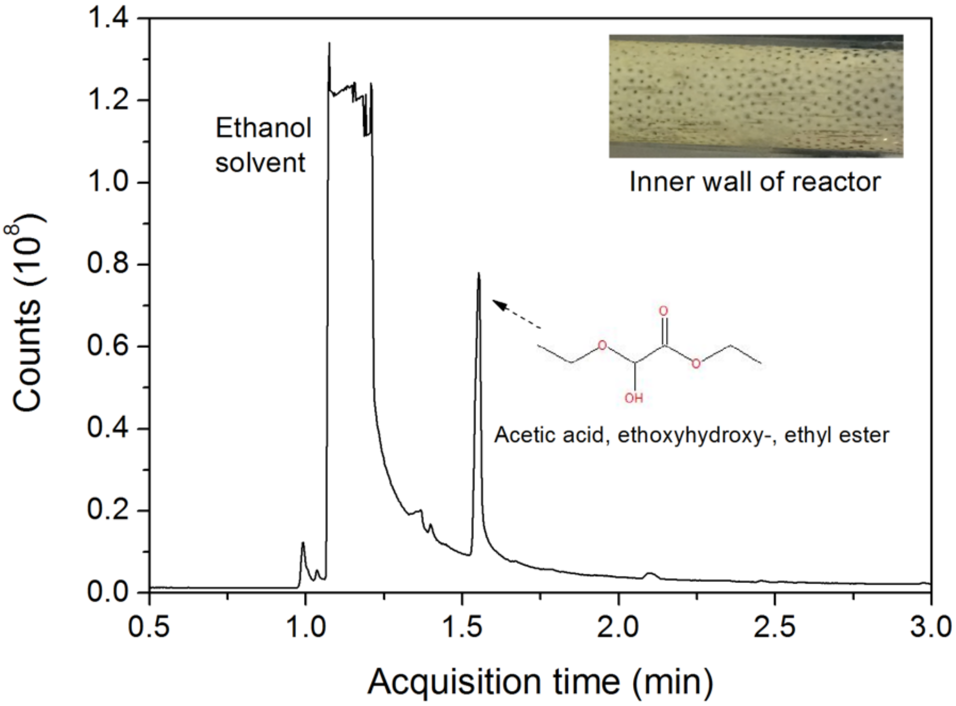

Figure S2. GC-MS analysis of the compound ($C_6H_{12}O_4$, CAS No.49653-17-0) formed on the internal surface of the DBD reactor (the compound was collected through dissolving using ethanol as the solvent).

Table S2. Reactive species produced in the CH_4/CO_2 discharge confirmed by optical emission spectra

Species	Wavelength (nm)	Transition	Reference
CH	431.4	$A^2\Delta \rightarrow X^2\Pi$;	[4]
C_2	516.5	$a^3\Pi_g \rightarrow d^3\Pi_g$ (0,0), $\Delta v = 0$	[5]
	563.5	$a^3\Pi_g \rightarrow d^3\Pi_g$ (1,0), $\Delta v = -1$	[5]
CO_2^+	353.2	$A^2\Pi \rightarrow X^2\Pi$	[6]
CO_2	391.7	$^1B_2 \rightarrow X^1\Sigma^+$	[6]
CO Angstrom band	451-608 nm	$B^1\Sigma \rightarrow A^1\Pi$,	[7]
H_α	656.3	$3d^2D \rightarrow 2p^2P^0$	[8]
O	777.5	$3s^5S^0 \rightarrow 3p^5P$	[9]
	844.7	$3s^3S^0 \rightarrow 3p^3P$	[9]

Table S3. Possible reactions in the CH_4/CO_2 DBD

Species	No.	Elementary reaction	Rate coefficient calculated from the Arrhenius expression, cm^3 $molecule^{-1}$ s^{-1}	E_a, kJ/mol
CO	S1	$CO_2 + e \rightarrow CO + O + e$	-	530.7 (5.5 eV)
	S2	$CO_2(v) + O \rightarrow CO + O_2$	-	48.3-96.5 (0.5-1 eV)
	S3	$CH + O \rightarrow CO + H$	6.59E-11	0
CH_3	S4	$CH_4 + e \rightarrow CH_3 + H + e$	-	849.2 (8.8 eV)
	S5	$CH_4 + OH \rightarrow CH_3 + H_2O$	6.68E-15	10.24

	S6	$CH_4 + O \rightarrow CH_3 + OH$	5.90E-18	28.06
	S7	$CH_4 + H \rightarrow CH_3 + H_2$	8.43E-19	33.59
OH	S8	$CH + O \rightarrow C + OH$	9.05E-15	19.79
	S9	$H_2 + O \rightarrow OH + H$	2.22E-17 [a]	12.14
	S10	$CO_2 + H \rightarrow OH + CO$	1.4E-29	111
	S11	$H + O_2 \rightarrow OH + O$	1.87E-22	70.34
	S12	$H + O \rightarrow OH$	4.33E-32 [b]	0
	S13	$H_2 + O_2 \rightarrow OH$	6.16E-62	292
CH_3CO	S14	$CH_3 + CO \rightarrow CH_3CO$	8.23E-18	28.77
CH_3COOH	S15	$CH_3CO + OH \rightarrow CH_3COOH$	-	0
	S16	$CH_3 + COOH \rightarrow CH_3COOH$	-	0
COOH	S17	$CO + OH \rightarrow COOH$	1.5E-12 [c]	2.4
	S18	$CO_2 + H \rightarrow COOH$	-	107.8
CH_3OH	S19	$CH_3 + OH \rightarrow CH_3OH$	9.84E-11	-0.14
C_2H_5	S20	$CH_3 + CH_3 \rightarrow C_2H_6$	6.03E-11	0
	S21	$C_2H_6 + OH \rightarrow C_2H_5 + H_2O$	2.55E-13	8.6
	S22	$C_2H_6 + O \rightarrow C_2H_5 + OH$	5.11E-16	24.28
	S23	$C_2H_6 + H \rightarrow C_2H_5 + H_2$	4.96E-17	31.01
C_2H_5OH	S24	$C_2H_5 + OH \rightarrow C_2H_5OH$	9.34E-11	-0.32

Reaction conditions for determining the rate coefficient: 1 atm, 298-300 K; [a] 320 K; [b] $cm^6/molecule^{-2}$ s^{-1}; [c] 400 K.

Plasma Simulation

The simulation employs a 0-dimension time-evaluated model using ZDplaskin. We assumed that no surface reactions and recirculation occurred in the DBD reactor so that all species could satisfy the conditions for solving the Boltzmann Equation (BE) (8).

$$\frac{\partial f}{\partial t} + v \cdot \nabla f - \frac{q}{m} E \cdot \nabla \mathrm{v} f = C[f] \quad (8)$$

Where E is the electric field，m is the electron mass, f is the electron energy distribution function (EEDF), q is the elementary charge, v is the average electron velocity and $C[f]$ represents the change rate of the EEDF. Our model can be classified into three main blocks; 263 electron-impact reactions, including momentum transfer, excitations/de-excitations, dissociation and ionisation reactions; 348 neutral-neutral reactions; 65 ion-neutral/radical/ion reactions. All simulated species are shown in table S4.

Table S4. Summary of all species included in the model.

Ground-state Neutrals & Radicals	CH_4 CH_3 CH_2 CH C C_2H_6 C_2H_5 C_2H_4 C_2H_3 C_2H_2 C_2H C_2 C_3H_8 C_3H_7 C_3H_6 C_3H_5 C_3H_4 C_3H_2 C_4H_2 C_4H_{10} CO_2 CO H_2 H O_2 O H_2O HO_2 OH H_2O_2 CH_2O CH_3CHO CH_2CHO CH_2CO C_2HO CH_3OH CH_3CO CH_2OH CH_3O HCO CH_3O_2 CH_3COOO C_2H_5OO CH_3COOOH CH_3COOH C_2H_5OH CH_3CHOH CH_3COCH_3 HCOOH COOH CH_3COCH_2 CH_3COO
Excited Neutrals & Radicals	$CH_4(v)$ $CO_2(v_{1-8})$ $CO_2(e_{1\&2})$ $C(^1D)$ $C(^1S)$ $CO(v_{1-10})$ $CO(e_{1-5})$ $H_2(j_{0-2})$ $H_2(j_{1-3})$ $H_2(v_{1-3})$ $H_2(e_{1\&2})$ $H_2(\Sigma)$… $H_2(\Pi)$ $O_2(v_{1-4})$ $H_2(r_{1\&2})$ $O(^1D)$ $O(^1S)$ $H_2O(v_{1-3})$ $C_2H_2(v_{2,5\&31})$ $C_2H_2(e_{1\&2})$ $C_2H_4(v_{1\&2})$ $C_2H_4(e_{1\&2})$ $C_2H_6(v_{13\&24})$ $C_3H_6(v)$ $C_3H_6(v_{1\&2})$ $C_3H_6(e)$
Charged Species	CH_4^+ CH_3^+ CH_2^+ CH^+ C^+ C_2^+ $C_2H_6^+$ $C_2H_6^+$ $C_2H_6^-$ $C_2H_5^+$ $C_2H_4^+$ $C_2H_3^+$ $C_2H_2^+$ C_2H^+ $C_3H_8^+$ $C_3H_8^-$ $C_3H_6^+$ $C_3H_6^-$ O_2^+ O_2^- O^+ O^- H_2^+ H^+ H^- H_2O^+ CO_2^+ CO^+ OH^-

The time evolution density of all species, $N_{i=1\ldots i\max}$, can be written as equation (9). The source term Q_{ij} is used to describe the contribution from each diverse reaction, j =1…$j_{\max}$, and it is defined by user's input file.

$$\frac{d[N_i]}{dt} = \sum_{j=1}^{j_{\max}} Q_{ij}(t) \tag{9}$$

In order to provide a better understanding of our physical model, we use reaction (10) as an example. The reaction rate of this reaction can be calculated using equation (11). Therefore, the source terms will be expressed as equation (12).

$$aA + bB \rightarrow a'A + cC \tag{10}$$

$$R = k_j[A]^a[B]^b \tag{11}$$

$$\mathrm{Q}_A = (a' - a)R;\ Q_B = -bR;\ Q_C = cR \tag{12}$$

Additionally, calculations of the rate constants, k_j (cm^3/s), are different if electrons are taken into account. For the neutral-neutral reactions, the rate constants can be obtained from the three-parameter Arrhenius form (13)

$$k_j(T) = A_j T^{B_j} \exp\left(-\frac{E_j}{RT}\right) \tag{13}$$

T is gas temperature in Kelvin. The three parameters of A_j, B_j and E_j represent pre-exponential factor, temperature factor, and activation energy respectively. All parameters used in this work can be found from NIST database.

However, for the electron-impact reactions, a special range of *E/n* was used to solve BE (8) to obtain the electron distribution function and mean electron temperature, while the rate constants for all electron-impact reactions were calculated using equation (14).

$$k = G\int_0^{\infty} \varepsilon\sigma_k F d\varepsilon \tag{14}$$

Where σ_k is the cross-section of the target particle, *F* represents the EEDFs, and ε (ε =v/G) is the electron energy in volt ($G = \sqrt{2e/m}$).

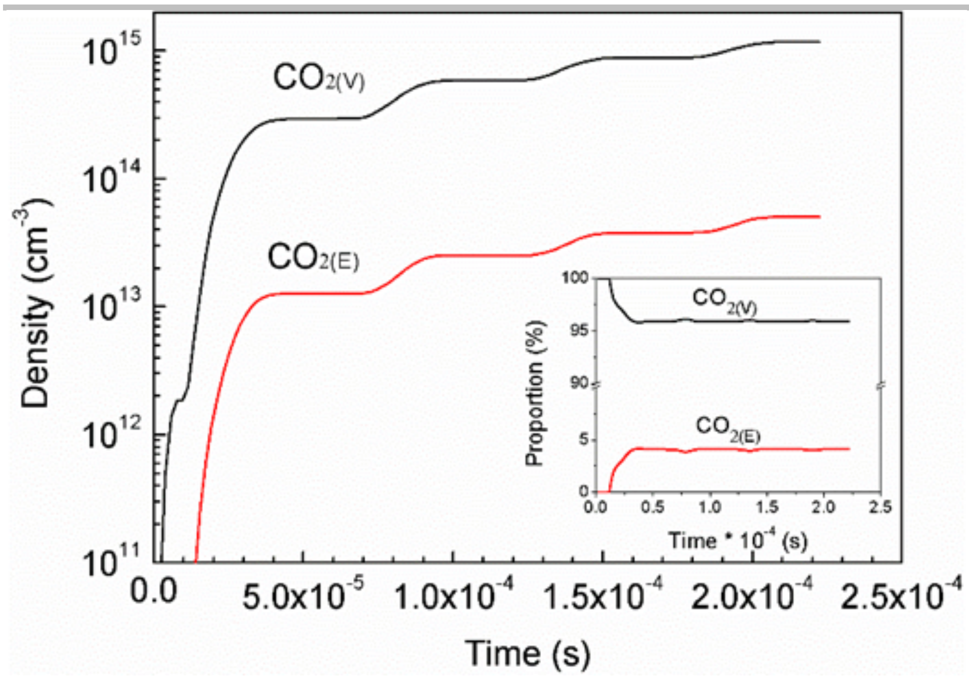

Figure S3. Temporal dynamic densities of vibrational and electronic excited CO_2 in two AC cycles (discharge frequency 9 kHz, The value of *E*/*n* is up to around 120 Td, and the electron energy distribution function (mean electron energy) is calculated using the Boltzmann Equation [10]. All the cross section data used for solving BE was from the LXCAT database (Morgan database), http://www.lxcat.net, retrieved on June 12, 2016).

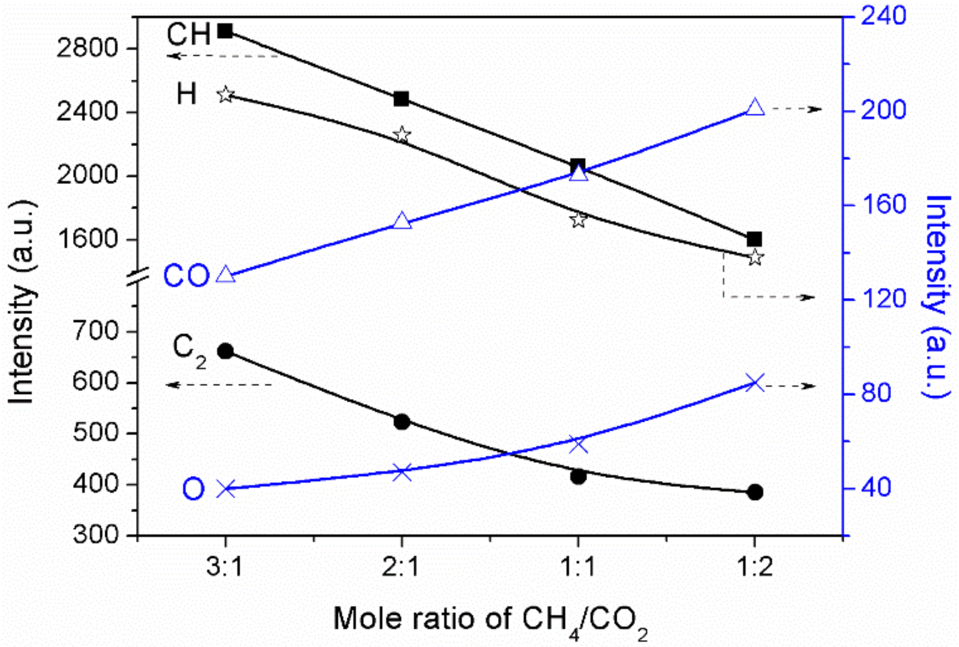

Figure S4. Effect of CH_4/CO_2 molar ratio on the relative intensity of different species in the CH_4/CO_2 discharge (CH 431.4 nm, H 656.3 nm, C_2 516.5 nm, CO 519.4 nm, O 844.7 nm, total flow rate 40 ml/min, discharge power 10 W, discharge frequency 9 kHz, 2 s exposure time).

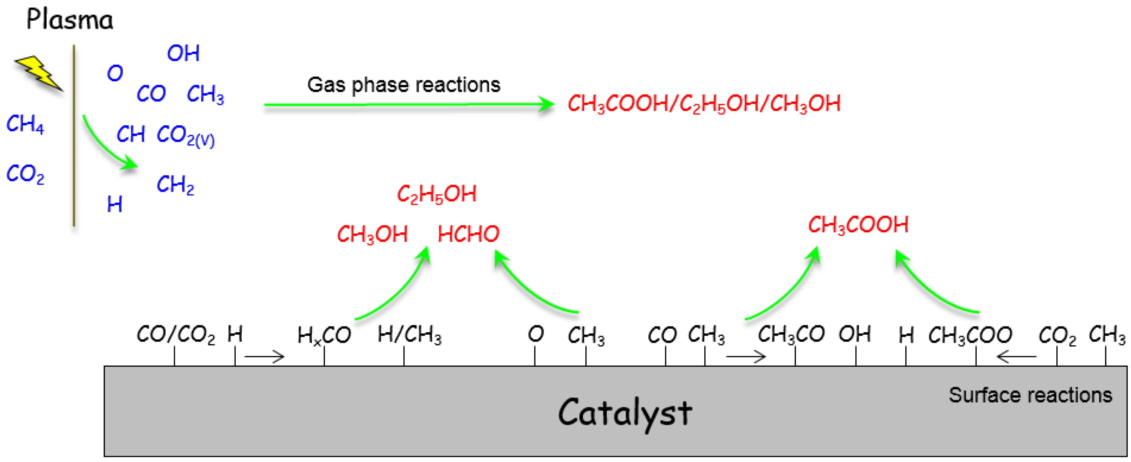

Scheme S3. Possible reaction mechanisms for the formation of CH_3COOH, CH_3OH, C_2H_5OH and HCHO using the plasma-catalysis approach.

The physicochemical properties of the support and catalysts were analyzed by means of N_2-physisorption, NH_3-TPD, XRD, H_2-TPR and TEM (Figure S5-S8) to understand the different reaction performance of γ-Al_2O_3 supported Cu, Au and Pt catalysts (Figure 1). The γ-Al_2O_3 support had a specific surface area of 114.8 m^2/g and plenty of acid sites (Figure S5); Cu, Au and Pt were highly dispersed on the surface of γ-Al_2O_3 with an average nanoparticle size of approx. 10 nm, 5 nm, and 5 nm, respectively (Figure S6); Cu existed in the form of CuO over γ-Al_2O_3 support with a reduction temperature in the range of 150-300 ^{o}C (Figure S7 and S8); Metallic Au (Au^0) and $Au^{\delta+}$ coexisted in the Au/γ-Al_2O_3 catalyst, as indicated by XRD and the TPR profile of the Au/γ-Al_2O_3 with reduction peak in the range of 310-400 ^{o}C (Figure S7 and S8); No diffraction peaks of Pt were observed on the XRD profile of the Pt/γ-Al_2O_3, caused by a high dispersion and small particle size for the Pt/γ-Al_2O_3 catalyst as confirmed by TEM (Figure S6 and S7).

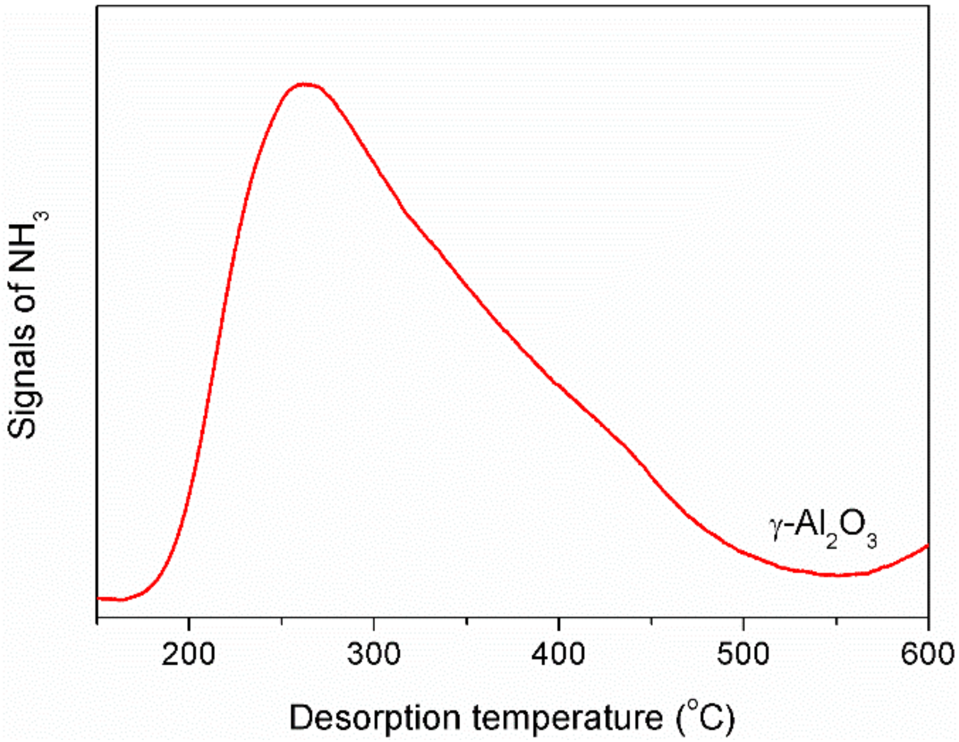

Figure S5. NH_3 temperature-programmed desorption profile of γ-Al_2O_3 support.

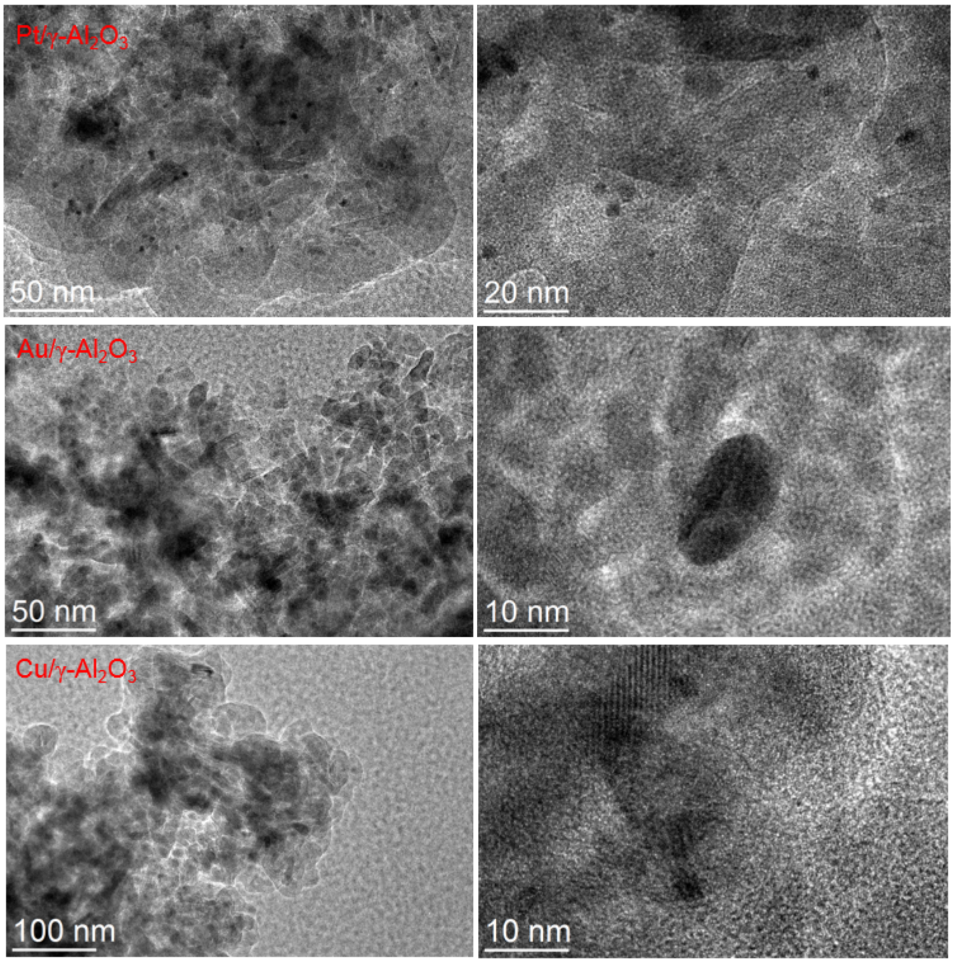

Figure S6. TEM images of as-synthesized catalysts.

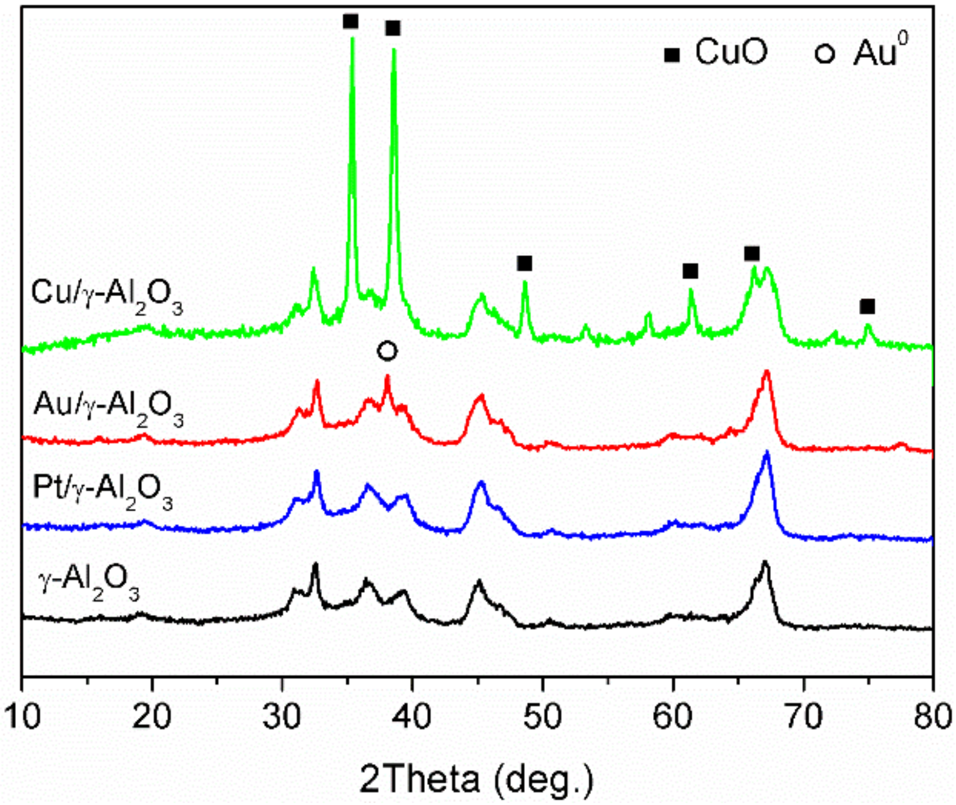

Figure S7. XRD patterns of as-synthesized catalysts (CuO, ICCD: 41-254).

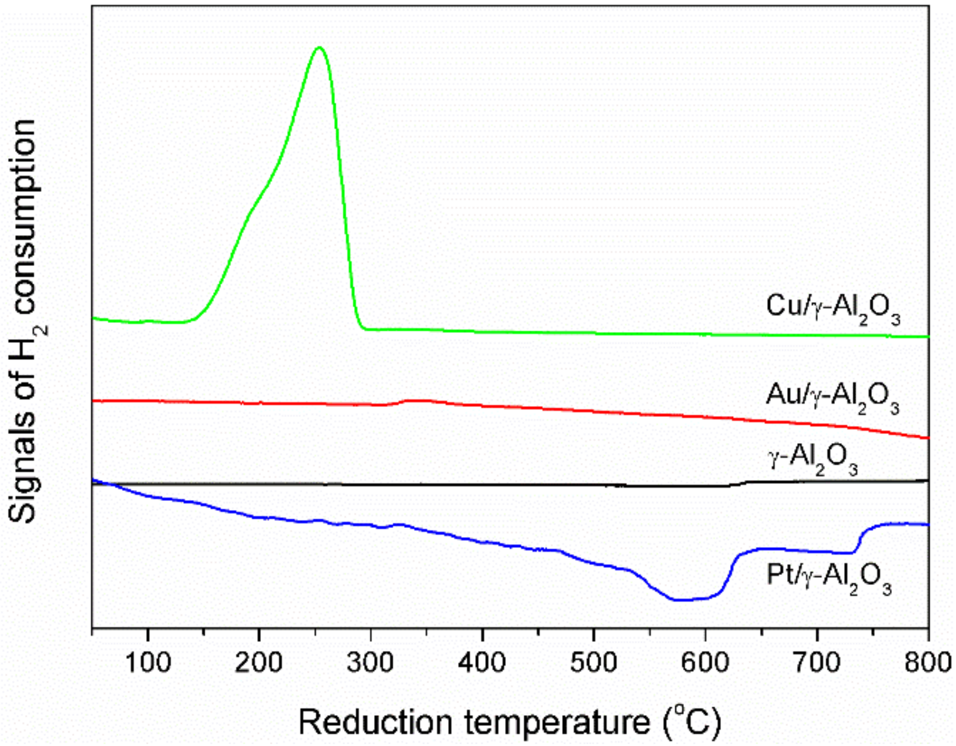

Figure S8. H_2 temperature-programmed reduction profiles of as-synthesized catalysts.

References

[1] X. Zhu, X. Gao, C. Zheng, Z. Wang, M. Ni, X. Tu, *RSC Advances* **2014**, *4*, 37796-37805.

[2] a) X. Tu, H. J. Gallon, M. V. Twigg, P. A. Gorry, J. C. Whitehead, *Journal of Physics D: Applied Physics* **2011**, *44*, 274007; b) X. Tu, J. Whitehead, *Applied Catalysis B: Environmental* **2012**, *125*, 439-448.

[3] a) H. K. Song, J.-W. Choi, S. H. Yue, H. Lee, B.-K. Na, *Catalysis Today* **2004**, *89*, 27-33; b) K. Zhang, B. Eliasson, U. Kogelschatz, *Industrial & Engineering Chemistry Research* **2002**, *41*, 1462-1468; c) B. Eliasson, C.-J. Liu, U. Kogelschatz, *Industrial & Engineering Chemistry Research* **2000**, *39*, 1221-1227.

[4] a) X. Wu, C. Li, Y. Wang, Z. Wang, C. Feng, H. Ding, *Applied Physics B* **2015**, *120*, 659-666; b) A. Yanguas-Gil, K. Focke, J. Benedikt, A. Von Keudell, *Journal of Applied Physics* **2007**, *101*, 103307-103500.

[5] J. Ma, M. N. Ashfold, Y. A. Mankelevich, *Journal of Applied Physics* **2009**, *105*, 043302.

[6] P. Reyes, E. Mendez, D. Osorio-Gonzalez, F. Castillo, H. Martínez, in *Physica Status Solidi C Conference, Vol. 5*, John Wiley & Sons, Ltd, **2008**, p. 907.

[7] A. Le Floch, C. Amiot, *Chemical Physics* **1985**, *97*, 379-389.

[8] Y. Yi, J. Zhou, H. Guo, J. Zhao, J. Su, L. Wang, X. Wang, W. Gong, *Angewandte Chemie* **2013**, *125*, 8604-8607.

[9] J. McConkey, C. Malone, P. Johnson, C. Winstead, V. McKoy, I. Kanik, *Physics Reports* **2008**, *466*, 1-103.

[10] G. Hagelaar, L. Pitchford, *Plasma Sources Science and Technology* **2005**, *14*, 722.
